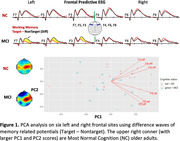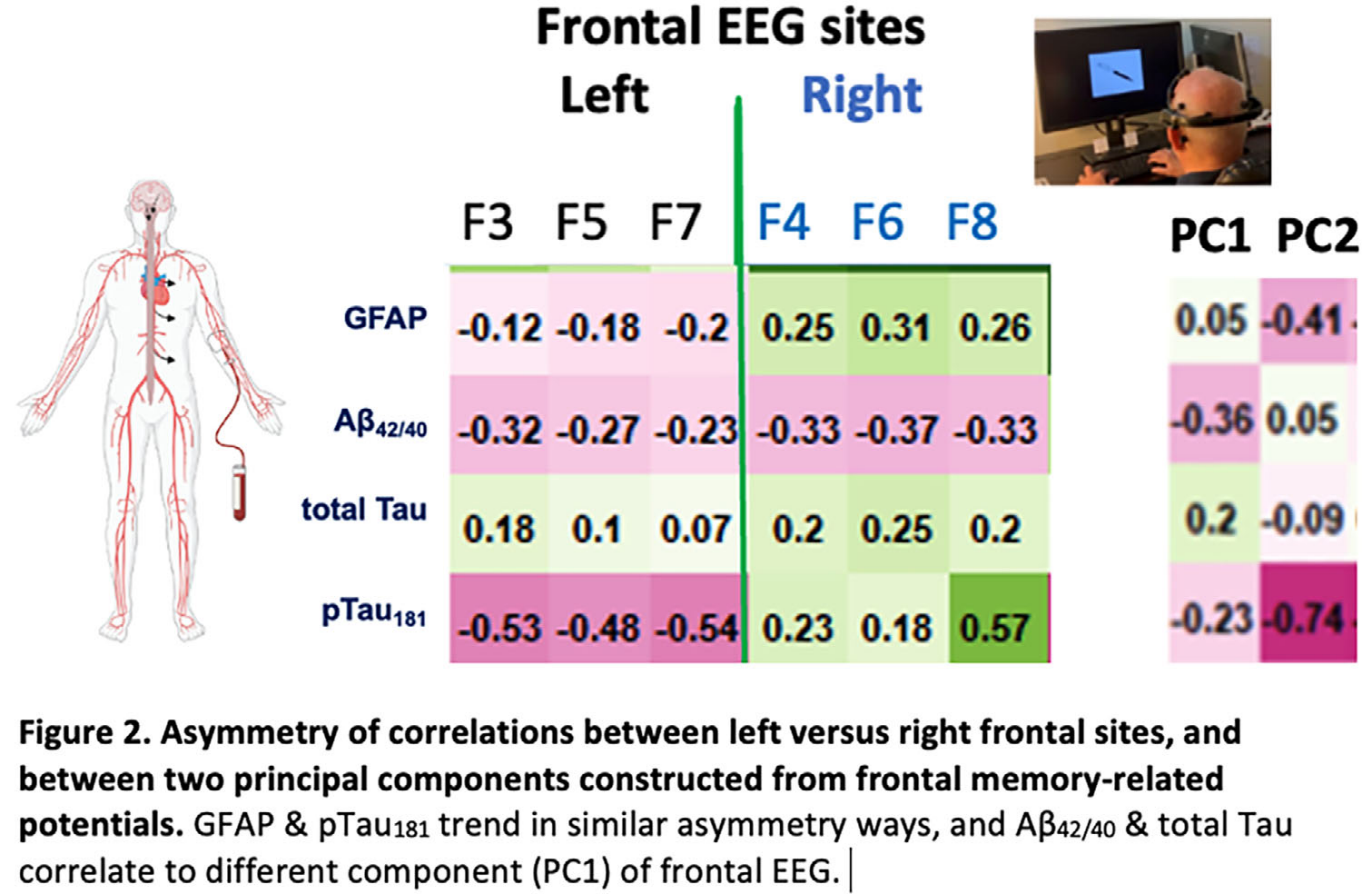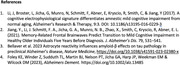# Frontal Memory‐related Brainwaves Differentially Correlate with AD and Astrocyte Plasma Biomarkers

**DOI:** 10.1002/alz.094185

**Published:** 2025-01-09

**Authors:** Yang Jiang, Xian Wu, Yuriko Katsumata, Maria F Clark, Kate E. Foley, Baoxi Wang, Tiffany L. Sudduth, Donna M. Wilcock, Gregory A Jicha, Christopher M. Norris

**Affiliations:** ^1^ University of Kentucky College of Medicine, Lexington, KY USA; ^2^ Sanders‐Brown Center on Aging, University of Kentucky, Lexington, KY USA; ^3^ Unviersity of Kentucky College of Medicine, Lexington, KY USA; ^4^ Indiana University School of Medicine/Stark Neurosciences Research Institute, Indianapolis, IN USA; ^5^ Jiangxi Normal University, Nanchang China; ^6^ University of Kentucky / Sanders‐Brown Center on Aging, Lexington, KY USA; ^7^ Indiana University School of Medicine, Stark Neurosciences Research Institute, Department of Neurology, Indianapolis, IN USA; ^8^ University of Kentucky College of Medicine, Sanders‐Brown Center on Aging, Lexington, KY USA

## Abstract

**Background:**

We currently lack in the dementia field accurate, noninvasive, quick, and affordable screening tools for brain dysfunctions associated with early subtle risk of mild cognitive impairment (MCI). Our Kentucky aging cohort demonstrates that asymptomatic older individuals with MCI‐like frontal memory‐related brainwave patterns convert to MCI within a short 5‐year period, as opposed to individuals with NC‐like patterns (1) that remain normal 10 years later (2). Astrocyte reactivity influences amyloid‐ß effects on tau pathology in preclinical Alzheimer’s disease (3). Leveraging blood‐based AD and astrocyte biomarkers and the cognitive electroencephalogram (EEG) signatures (4), we test the hypothesis that predictive frontal memory‐related EEG changes correlate with preclinical and early AD plasma biomarkers.

**Method:**

34 (19 women) older volunteers with or without MCI, average age 79 (SD 8.53) years old, from a longitudinal cohort followed by University of Kentucky ADRC participated. Each participant’s EEG was recorded (64‐ or 14‐channels) during a working memory (modified delayed match‐to‐sample) task. Principal component analysis (PCA) was performed on 64‐channel EEG data to create PC scores (PC1 & PC2). For multiple linear regression of EEG PC scores on multiple neurodegenerative plasma biomarkers including Aß42/40, pTau181, total Tau, and GFAP (Astrocyte reactivity), we adjusted age, sex, education, and gap years between collection dates.

**Result:**

The 61% of variance in frontal signals can be explained by PC1 in normal cognition (NC) and MCI individuals, and PC2 counts for 35% of variance (Figure 1). The decreased brainwaves (MCI‐like) seen in left frontal sites significantly correlate with increased pTau181, GFAP, and PC2 (Figure 2). Curiously, right frontal EEG relations with pTau181, GFAP showed the opposite trend. Bilateral frontal signals showed negative correlations with Aß42/40 and positive correlations with total Tau.

**Conclusion:**

Our results indicate that GFAP & pTau181 trend in similar asymmetry ways with frontal cognitive brainwaves, but Aß42/40 & total Tau correlate to a different component of frontal EEG. That is, distinct cognitive brainwaves correlate with astrocyte reactivity differentially that influence pathologies of beta‐amyloid accumulations and Tau development. Cognitive pathophysiological signatures and AD–Astrocyte plasma biomarkers have great potential for predicting subtle cognitive decline and specific dementia risk in healthy normal individuals.